# 

**Published:** 1889-08

**Authors:** 


					﻿JLTJG-TJST, 1869.
OLD SERIES
Vol. XXV
NEW SERIES
Vol. VI.—No. 6.
4^
1855 o


Journal
SOS


W. F. WESTMORELAND, M.D,'
H. V. M. MILLER, M. D.LL. D.<
VIRGIL 0. HARDON, M. D.
MlBUSHED By JAMES P.HARRISON&cd
W ______________jgTLANTA.GA.
SUBSCRIPTIONS S2.6O A YEAR
				

